# Davunetide promotes structural and functional recovery of the injured spinal cord by promoting autophagy

**DOI:** 10.4103/NRR.NRR-D-24-00154

**Published:** 2025-03-25

**Authors:** Yituo Chen, Rongjie Liu, Wanta Cai, Liting Jiang, Kongbin Chen, Jingwei Shi, Junsheng Lou, Letian Yu, Chenyu Wu, Liangliang Yang, Kailiang Zhou, Wenfei Ni

**Affiliations:** 1 Department of Orthopedics, The Second Affiliated Hospital and Yuying Children’s Hospital of Wenzhou Medical University, Wenzhou, Zhejiang Province, China; 2 Zhejiang Provincial Key Laboratory of Orthopedics, Wenzhou, Zhejiang Province, China; 3 The Second Clinical Medical College of Wenzhou Medical University, Wenzhou, Zhejiang Province, China; 4 Cixi Biomedical Research Institute, Wenzhou Medical University, Ningbo, Zhejiang Province, China; 5 Department of Orthopedic Surgery, The First Affiliated Hospital, Zhejiang University School of Medicine, Hangzhou, Zhejiang Province, China; 6 Renji College of Wenzhou Medical University, Wenzhou, Zhejiang Province, China; 7 School of Pharmaceutical Sciences, Wenzhou Medical University, Wenzhou, Zhejiang Provine, China

**Keywords:** autophagy, davunetide, locomotor function recovery, NAP, necroptosis, neuron, Sirtuin1, spinal cord injury, transcription factor EB

## Abstract

After spinal cord injury, programmed cell death is common. In this context, autophagy plays a crucial role in clearing cellular debris, while necroptosis exacerbates neuroinflammation and further damages neural structures. The neuroprotective drug davunetide has shown substantial therapeutic effects on brain diseases, but its role in treating spinal cord injury remains unclear. Therefore, the aim of this study was to investigate the effects of davunetide on cell death after spinal cord injury. To do this, we established a mouse model of spinal cord contusion and administered davunetide intranasally daily at a dose of 0.5 µg/5 µL. Mouse locomotor function was assessed using footprint analysis and Basso Mouse Scale scoring, while the extent of spinal cord injury was evaluated using Masson’s trichrome staining. The expression levels of proteins related to locomotor function and spinal cord injury were analyzed by Western blotting and immunofluorescence staining, and protein–protein interactions were evaluated using immunoprecipitation techniques. Our results demonstrated that davunetide not only reduced the size of the injury area but also promoted the recovery of locomotor function after spinal cord injury. Specifically, davunetide exerted its effects by enhancing autophagy and inhibiting necroptosis. Inhibition of autophagy reversed the protective effects of davunetide on necroptosis. Further investigation revealed that davunetide acted through the SIRT1-FOXO1-TFEB signaling pathway, which is key to its therapeutic effects. These findings suggest the potential of davunetide in the treatment of spinal cord injury and provide valuable insights into the underlying mechanisms. This study offers strong scientific evidence to support the development of new therapeutic strategies for spinal cord injury.

## Graphical abstract

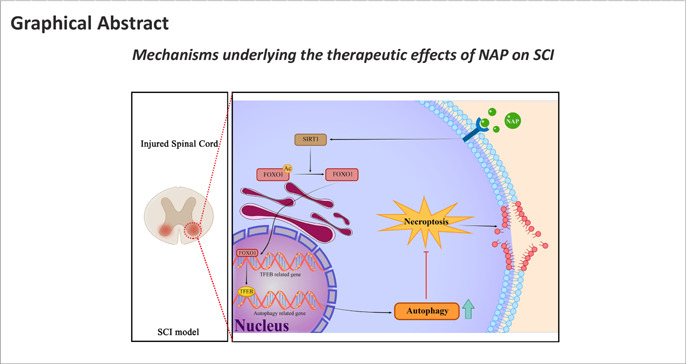

## Introduction

Spinal cord injury (SCI) represents a critical health challenge due to its association with severe disability and mortality (Sutor et al., 2022). SCI encompasses both initial and subsequent injury phases, with the latter being more prolonged and consequential. This phase often leads to oxidative stress, mitochondrial dysfunction, inflammation, and cellular demise (Ni et al., 2022). Emerging research indicates that programmed cell death, incorporating newly identified forms such as necroptosis, autophagy, and pyroptosis, plays a pivotal role in SCI progression (Shi et al., 2021). Consequently, therapeutic strategies targeting cellular death mechanisms are gaining prominence in SCI research. Despite the lack of established clinical treatment protocols for SCI, modulating cell death pathways is increasingly recognized as a promising therapeutic avenue.

Traditionally, necrosis was perceived as an unregulated form of cell death. However, recent studies have identified a regulated form of necrosis, referred to as “necroptosis” (Su et al., 2015; Shi et al., 2021). This process, initiated by extracellular signals like TNF-α, involves the formation of a necrosome through assembly of the receptor-interacting protein kinase 1 (RIP)/receptor-interacting protein kinase 3 (RIP3) complex and subsequent phosphorylation of mixed lineage kinase domain-like protein (MLKL). This leads to cellular disruption (Bertheloot et al., 2021). In the context of SCI, necroptosis intensifies the secondary injury, but inhibition of necroptosis has been shown to facilitate spinal cord repair and regeneration post-injury (Fiani et al., 2021). Therefore, targeting necroptosis in neuronal cells has emerged as a promising approach for SCI treatment. In contrast to necroptosis, autophagy (a downregulatory mechanism that is aimed at recycling structural components of the cell) promotes cellular stability. This process helps degrade faulty cellular organelles and harmful byproducts of protein metabolism through the autophagosomal-lysosomal pathway (Shi et al., 2021). Previous research indicates that autophagy impairment in SCI exacerbates neuronal damage and inflammation (Zhang et al., 2023a). Enhancing autophagy in neuronal cells not only aids in the recovery of motor functions but also reduces neuron loss (Li et al., 2022; Zhang et al., 2023b). Due to its role in removing damaged organelles and abnormal proteins, autophagy may be crucial to maintaining neuronal stability and self-renewal after SCI. Moreover, it has been shown that an inverse relationship between autophagy and necroptosis, as promoting autophagy inhibits necroptosis after SCI (Chen et al., 2024). In addition, some necroptosis markers such as MLKL are cleared through the autophagy pathway, and blockade of autophagic flux can inhibit MLKL clearance, thereby exacerbating necroptosis (Liu et al., 2018). Therefore, autophagy acts as a neuroprotective mechanism by modulating neural cell death after SCI.

SIRT1, an important member of the sirtuin family, is a Class III histone deacetylase that participates in glucose metabolism, cell survival, mitochondrial respiration regulation, and autophagy (Chen et al., 2022). Members of the mammalian SIRT protein family can interact with p53, FOXO, PGC-1α, NF-κB, Ku70, and other proteins to regulate the cellular stress response, thereby affecting biological processes such as cell metabolism, aging, and apoptosis (Xie et al., 2022). Furthermore, SIRT1 regulates autophagy by deacetylating the autophagy-related genes *Atg5*, *Atg7*, and *Atg8* (Lee et al., 2008). Thus, SIRT1-regulated autophagy may promote SCI repair. SIRT1 has several positive effects on SCI, such as counteracting oxidative stress, reducing cell death, and diminishing disruption of the blood–spinal cord barrier (Yu et al., 2019; Gao et al., 2020; Jiang et al., 2023). Consequently, SIRT1’s neuroprotective properties in the aftermath of SCI have been established, positioning it as a promising therapeutic agent for SCI management.

Transcription factor EB (TFEB) regulates autophagy by binding to the promoter regions of autophagy-/lysosomal degradation-related genes (Liang et al., 2023). This regulatory mechanism enables TFEB to precisely regulate autophagy at the transcriptional level. By binding to their promoter regions, TFEB enhances the expression of genes related to autophagy and lysosome biogenesis, thereby promoting autophagy progression (Settembre et al., 2011). In response to multiple signaling molecules, TFEB translocates into the nucleus and regulates gene expression, a process that largely depends on its altered phosphorylation status (Zhang et al., 2023b). Previous studies have shown that nuclear TFEB promotes axonal regeneration, inhibits glial scar formation, and fosters functional recovery by enhancing autophagosome formation and restoring autophagic flux following SCI (Lu et al., 2023; Xu et al., 2023b). Overall, TFEB, as the main regulatory factor of autophagy, ensures normal progression of autophagy by regulating the transcription levels of autophagy-/lysosome-related genes, and is thus a promising therapeutic target for promoting SCI repair.

Davunetide (NAP), a peptide consisting of eight amino acids and originating from the activity-dependent neuroprotective protein (ADNP), exerts neuroprotective properties in the context of central nervous system injuries (Magen and Gozes, 2014). Earlier studies demonstrated that NAP stabilizes microtubules and reduces tau phosphorylation, and thus inhibits the progression of neurodegenerative diseases (Shiryaev et al., 2009; Zhang et al., 2017). In addition, NAP has anti-inflammatory and antioxidant capabilities, prevents protein aggregation, and modulates cell death. These properties suggest that NAP plays a crucial role in addressing central nervous system trauma (Magen and Gozes, 2014; Zhang et al., 2017). Most importantly, ADNP (the precursor of NAP) can interact with SIRT1 through the microtubule-end binding proteins EB1/EB3 and thus play an important role in mitochondrial energy metabolism linked to autophagy stress responses (D’Incal et al., 2024). SIRT1 is a key regulatory factor that indirectly regulates TFEB by regulating different signaling pathways such as the mTOR signaling pathway, thereby affecting the level of autophagy that occurs after SCI (Lu et al., 2021). Therefore, NAP could be useful in treating SCI, particularly because it promotes functional recovery by regulating autophagy through the SIRT1-TFEB pathway. However, few studies have explored the role of NAP in SCI. Given the critical role of cell death in SCI pathophysiology, the aim of the current study was to explore the effects of NAP on motor function post-SCI and to determine whether these effects are related to autophagy and necroptosis. This study underscores the potential of NAP as a promising therapeutic agent for SCI.

## Methods

### Mouse spinal cord contusion model

In this study on SCI, only female mice were used because of the anatomical convenience of their shorter urethras for facilitating artificial voiding, a necessity to prevent urinary retention post-SCI. In male mice, the extended length of the urethra complicates such procedures (Wang et al., 2023). Healthy female C57BL/6 mice (6–8 weeks old, body weight 25–30 g) were sourced from the Experimental Animal Center of Wenzhou Medical University, Zhejiang Province, China (license number SYXK (Zhe) 2021-0020).

To deliver the spinal cord contusion, first mice were anesthetized by intraperitoneal injection of 1% pentobarbital sodium (50 mg/kg, Shanghai Fuzhe Chemicals Ltd., Shanghai, China). Then, a T9–T10 laminectomy was performed from under the T7 to the top of the R3 paddle. A spinal impactor (1.2 mm diameter, 10 g weight) was then released from a 20 mm height, impacting directly onto the spinal cord’s exposed surface, inducing moderate SCI. Post-injury, the spinal cord’s integrity was meticulously inspected for hemorrhage or ruptures. Subsequently, the muscle layers, fascia, and skin were sutured in a stratified manner using 4-0 non-absorbable silk thread. In the sham surgery group, identical surgical procedures were performed without inflicting any weight-induced damage. After the procedure, the mice were supported with artificial urinary voiding three times a day and were maintained under standard housing conditions: temperature 21–25°C, light intensity 100 fc, 12/12-hour light/dark cycle, humidity 50%–60%, and free access to food and water. Every day after the surgery, iodine was used to disinfect the surgical wounds and urethras of the mice to prevent infection. All the animal experiments were carried out in accordance with the Guide for Care and Use of Laboratory Animals issued by the National Institutes of Health and approved by the Ethics Committee on Animal Experimentation, Wenzhou Medical University (approval No. xmsq 2022-0571). All experiments were designed and reported according to the Animal Research: Reporting of *In Vivo* Experiments (ARRIVE) guidelines (Percie du Sert et al., 2020).

### Adeno-associated virus vector packaging

Genechem Co., Ltd. (Shanghai, China) was responsible for the construction and packaging of adeno-associated virus (AAV)-SIRT1 shRNA (mouse shSIRT1) and AAV-TFEB shRNA (mouse shTFEB). The titers of the AAV-SIRT1 shRNA and AAV-TFEB shRNA vectors were determined to be 3.6 × 10^13^ and 1.0 × 10^13^ genomic copies per milliliter, respectively, as established through quantitative PCR (qPCR) analysis.

### Drug and adeno-associated virus vector administration

**Figure 1:**
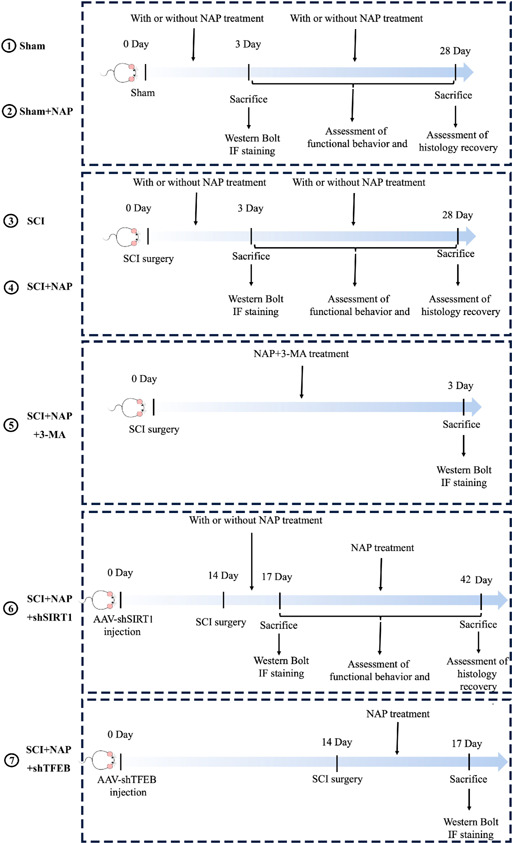
Animal experiment flow chart. First, 6- to 8-week-old mice were subjected to a Sham or SCI operation. After the operation, the animals were treated as indicated (groups 1–5). In some experiments, the animals were pre-treated AAV-shTFEB or AAV-shSIRT1 2 weeks before the surgery (groups 6 and 7). Animals were sacrificed on day 3 or 28 after injury for assessment. AAV: Adeno-associated virus; IF: immunofluorescence; NAP: davunetide; SCI: spinal cord injury; SIRT1: Sirtuin1; TFEB: transcription factor EB.

The group assignments and associated procedures and treatments are shown in **Figure 1**. In total, 220 mice were randomly assigned to seven distinct groups: Sham (*n* = 50), Sham + NAP (*n* = 20), SCI (*n* = 55), SCI + NAP (*n* = 60), SCI + NAP + 3MA (*n* = 5), SCI + NAP + shSIRT1 (*n* = 25), and SCI + NAP + shTFEB (*n* = 5). Intranasal administration was used in this study, as nasal blood vessels are abundant, drugs can be quickly absorbed into the bloodstream, and it is a noninvasive administration route that shows good bioavailability and pharmacokinetics (Gozes et al., 2000). NAP (Cat# 211439-12-2, MedChemExpress, Monmouth Junction, NJ, USA) was prepared in phosphate-buffered saline (PBS) and administered intranasally, replicating the methodology of prior studies, at a dosage of 0.5 µg/5 µL per day for 3 or 28 days (Merenlender-Wagner et al., 2010). 3-Methyladenine (3MA) (Cat# M9281, Sigma‒Aldrich, St. Louis, USA), dissolved in PBS, was administered intraperitoneally at a dose of 15 mg/kg (Zhang et al., 2023b), thirty minutes before NAP administration for 3 days. Two weeks before SCI, viral vectors in PBS were administered at the T9–T10 level to a depth of 1.5 mm from the dorsal surface of the spinal cord with microsyringes to mice in the SCI + NAP + shSIRT1 and SCI + NAP + shTFEB groups for either SIRT1 gene expression or TFEB suppression. For the next 14 days, these groups were treated like the SCI + NAP group. On days 3 and 28, some of the mice from each group were euthanized with an overdose of sodium pentobarbital (500 mg/kg), and samples were collected for histology.

### Functional behavioral assessment

Basso Mouse Scale (BMS) scores were employed to assess motor function in mice at various intervals (0, 1, 3, 7, 14, and 28 days) after spinal cord injury (Ni et al., 2022). The BMS scoring system ranges from 0, indicating total paralysis, to 9, denoting normal movement. Additionally, footprint analysis was conducted at 28 days post-surgery to further evaluate motor function. For this analysis, the hind limbs and forelimbs were marked with red and blue dyes, respectively. The results were independently evaluated by two blinded assessors who were unaware of the experimental conditions.

### Hematoxylin-eosin, Masson, and Nissl staining

Hematoxylin and eosin (HE) and Masson’s trichrome staining were conducted as per the methodologies outlined in a previous study (Xu et al., 2023b). At 28-days post-surgery, the mice were anesthetized using 1% (w/v) pentobarbital sodium (50 mg/kg, given intraperitoneally), and the spinal cord tissue was perfused with PBS. A15-mm section of the spinal cord centered around the injury epicenter was carefully extracted and subsequently preserved in 4% (w/v) paraformaldehyde for a 48-hour period. Following preservation, the spinal cord was embedded in paraffin, and 4-μm thick-longitudinal sections were cut using a precision microtome The slides bearing the tissue sections were subjected to degreasing, dehydration, and immersion in Masson’s trichrome stain (Cat# G1340, Solarbio Science & Technology, Beijing, China), followed by multiple distilled water washes, according to standardized histological procedures. For HE staining, a similar protocol of degreasing, dehydrating, and sequential immersion in hematoxylin and eosin dyes (Cat# G1120, Solarbio Science & Technology) was followed, interspersed with washing steps. After these procedures, the slides were carefully dehydrated and securely sealed. Nissl staining was performed using a Nissl Stain Kit according to the manufacturer’s protocol (G1430; Solarbio Science & Technology). Cross-sections of the spinal cord, taken 28 days post-operation, were subjected to deparaffinization and rehydration, followed by 10-minute staining with Cresyl Violet. The sections were then thoroughly rinsed with deionized water and differentiated using Nissl differentiation solution until the majority of the stain was eliminated. After a final dehydration step in xylene, the slides bearing these sections were sealed with neutral resin, rendering them ready for microscopic examination. Quantification of the stained areas within the Masson-stained lesions was performed using the thresholding method in ImageJ software 1.0 (National Institutes of Health, Bethesda, MD, USA; Schneider et al., 2012) in a double-blind manner. The result of lesion area is expressed as fold changes in comparison to the SCI group.

### Western blot analysis

Mice were euthanized 3 days post-SCI, and the spinal cord segment containing the injury site (1.5 cm, centering the injury epicenter) was harvested. These samples were stored at –80°C until required for Western blot analysis. For sample preparation, the spinal cord sections were placed in 1.5-mL tissue grinders with RIPA buffer, PMSF, and phosphatase inhibitors, incubated at room temperature for 10 minutes, and homogenized using a homogenizer. The tissue lysis solution was separated by centrifugation at 20,000 × *g* at 4°C for 30 minutes, and the supernatant was maintained on ice for subsequent analysis. Protein concentrations were determined by BCA assay, and equal amounts of protein (60 µg per lane) were subjected to 12.5% (w/v) SDS-PAGE. The samples were then transferred onto polyvinylidene fluoride (PVDF) membranes (Roche Applied Science, Indianapolis, IN, USA) that were blocked in 5% (w/v) skimmed milk and incubated overnight at 4°C with the following primary antibodies: Beclin-1 (anti-rabbit, 1:1000, Cat# 3738, Cell Signaling Technology, Danvers, MA, USA), SQSTM1/p62 (anti-mouse,1:1000, Cat# ab240635, Abcam, Cambridge, MA, USA), LC3B (anti-rabbit, 1:1000, Cat# 3868, Cell Signaling Technology), CTSD (anti-rabbit, 1:1000, Cat# 21327-1, Proteintech, Rosemont, IL, USA), RIP (anti-rabbit, 1:1000, Cat# 3493, Cell Signaling Technology), p-RIP (anti-rabbit, 1:1000, Cat# 31122, Cell Signaling Technology), RIP3 (anti-rabbit, 1:1000, Cat# 95702, Cell Signaling Technology), p-RIP3 (anti-rabbit, 1:1000, Cat# 91702, Cell Signaling Technology), MLKL (anti-rabbit, 1:1000, Cat# AF7919, Affinity, Shanghai, China), p-MLKL (anti-rabbit, 1:1000, Cat# AF7420, Affinity), SIRT1 (anti-rabbit, 1:1000, Cat# 8469, Cell Signaling Technology), TFEB (anti-rabbit, 1:1000, Cat# AF7015, Affinity), AC-FOXO1 (anti-rabbit, 1:1000, Cat# AF2305, Affinity), histone H3 (anti-rabbit, 1:1000, Cat# BF9211, Affinity), and β-actin (anti-rabbit, 1:1000, Cat# AF7018, Affinity). The membranes were then incubated with HRP-conjugated goat anti-mouse IgG (H+L) (Proteintech, Cat# SA00001-1) or HRP-conjugated goat anti-rabbit IgG (H+L) (Proteintech, Cat# SA00001-2) secondary antibodies at room temperature for 2 hours. Protein bands were visualized and analyzed using a ChemiDoc^TM^ XRS+ Imaging System (Bio-Rad, Hercules, CA, USA) with ECL. Protein expression was quantified using software ImageJ and normalized to β-actin.

### Immunofluorescence staining

Three- and twenty-eight-days post SCI, spinal cord specimens were excised from mice for immunofluorescence (IF) staining. Sections (1 mm in length, located 4 mm from the epicenter) were taken and subjected to dewaxing and rehydration. Subsequently, they were washed thrice in PBS to eliminate excess impurities and contaminants. The sections were then immersed in a container filled with 10.2 mM citrate buffer and heated at 95°C for 20 minutes, followed by treatment with a PBS solution containing Triton X-100 for cell membrane permeabilization. The sections were blocked with a 10% (v/v) bovine serum albumin solution in PBS for 1 hour. Then, the sections were incubated overnight at 4°C with antibodies directed against LC3B (anti-rabbit, 1:200, Cat# 3868, Cell Signaling Technology), neuron-specific nuclear protein (NeuN; anti-mouse, 1:400, Cat# ab104224, Abcam), ionized calcium-binding adapter molecule 1 (Iba-1; anti-mouse, 1:200, Cat# ab283319, Abcam), glial fibrillary acidic protein (GFAP; anti-mouse, 1:200, Cat# ab4648, Abcam), SQSTM1/p62 (anti-mouse, 1:200, Cat# ab2406, Abcam), p-MLKL (1:200, Cat# AF7420, Affinity), SIRT1 (anti-rabbit, 1:200, Cat# 8469, Cell Signaling Technology), TFEB (anti-rabbit, 1:200, Cat# AF7015, Affinity), neurofilament heavy polypeptide (NF200; anti-rabbit, 1:200, Cat# DF13211, Affinity), and synaptophysin (Syn; anti-rabbit, 1:200, Cat#32127, Abcam). Next, a meticulous triple wash with PBS was performed to ensure complete elimination of unbound antibodies and contaminants. This step was followed by incubation with fluorescein isothiocyanate-conjugated secondary antibodies including goat anti-mouse IgG H&L (Alexa Fluor® 594) (1:500, Cat# ab150116, Abcam), goat anti-mouse IgG H&L (Alexa Fluor® 488) (1:500, Cat# ab150113, Abcam), goat anti-rabbit IgG H&L (Alexa Fluor® 488) (1:500, Cat# ab150077, Abcam), and goat anti-rabbit IgG H&L (Alexa Fluor® 594) (1:500, Cat# ab150080, Abcam). Finally, the sections were stained with 4′,6-diamidino-2-phenylindole (DAPI; Beyotime Biotechnology, Jiangsu, China) for nuclear visualization. Detailed observations and assessments of the staining patterns were carried out using a high-resolution fluorescence microscope (Olympus, Tokyo, Japan). The immunofluorescence intensity of p62, MLKL, and SIRT1 in individual neurons was quantified using ImageJ software. In addition, a precise, double-blind manual counting of LC3 puncta and synaptic connections in each neuron was also undertaken, utilizing the same software.

### Bioinformatics

SCI-related targets were identified through GeneCards (https://www.genecards.org/) searches using key terms such as “spinal cord injury.” NAP-associated targets were extracted from SwissTargetPrediction (http://www.swisstargetprediction.ch/) and Pharmapper (http://www.lilab-ecust.cn/pharmmapper/), and these two lists were compared to ascertain the common targets. DAVID (https://david.ncifcrf.gov/) was used to perform Kyoto Encyclopedia of Genes and Genomes (KEGG) and GO analyses of the common target genes (Huang da et al., 2009). A protein-protein interaction (PPI) was constructed utilizing the STRING database (www.string-db.org/) (Szklarczyk et al., 2017). Network analysis of these interactions was conducted using Cytoscape v3.6.1 (https://cytoscape.org/).

### Immunoprecipitation

As described by Xu et al. (2023b), spinal cord tissue samples were homogenized in a solution containing 0.1 μM aprotinin 50 mM NaF, 1% IGEPAL CA-630, 150 mM NaCl, 10 mM EDTA, 1 μM leupeptin, and 50 mM Tris-HCl, then centrifuged, and the supernatant was subjected to immunoprecipitation. Utilizing the Pierce Crosslink Immunoprecipitation Kit (Elabscience, Wuhan, China), the primary antibody was covalently bonded to protein A/G agarose beads as per the instructions provided by the manufacturer. The bead-bound antibody was then incubated with the sample at 4°C for 2 hours. An IgG isotype control was used for comparative analysis. Following immunoprecipitation and subsequent washes with Tris-buffered saline (TBS), the proteins were eluted from the beads using glycine-HCl (0.1 M, pH 3.5) and subjected to protein immunostaining using an AC-lysine antibody (1:1000, Cat# sc-32268, Santa Cruz Biotechnology, Santa Cruz, CA, USA).

### SIRT activity assay

SIRT activity of spinal cord tissue lysis solution was measured by assay kit (Cat# ab156065, Abcam) according to manufactures’ instructions. The result is expressed as fold change relative to the Sham group.

### Statistical analysis

All statistical analyses were performed utilizing SPSS software, version 19 (SPSS, Chicago, IL, USA) in a double-blind manner. The data are expressed as mean ± SEM. All data were normalized to mitigate the impact of extraneous variables. Independent sample *t*-tests were used to compare two groups. Three or four groups with normally distributed data were compared by two-way analysis of variance, followed by Tukey’s multiple comparisons test. For data sets lacking normal distribution, the nonparametric Mann–Whitney *U* test was used. The significance threshold was set at a *P*-value of less than 0.05. For robust statistical analysis, a minimum sample size of five animals per group was used.

## Results

### Davunetide promotes motor function recovery after spinal cord injury

**Figure 2:**
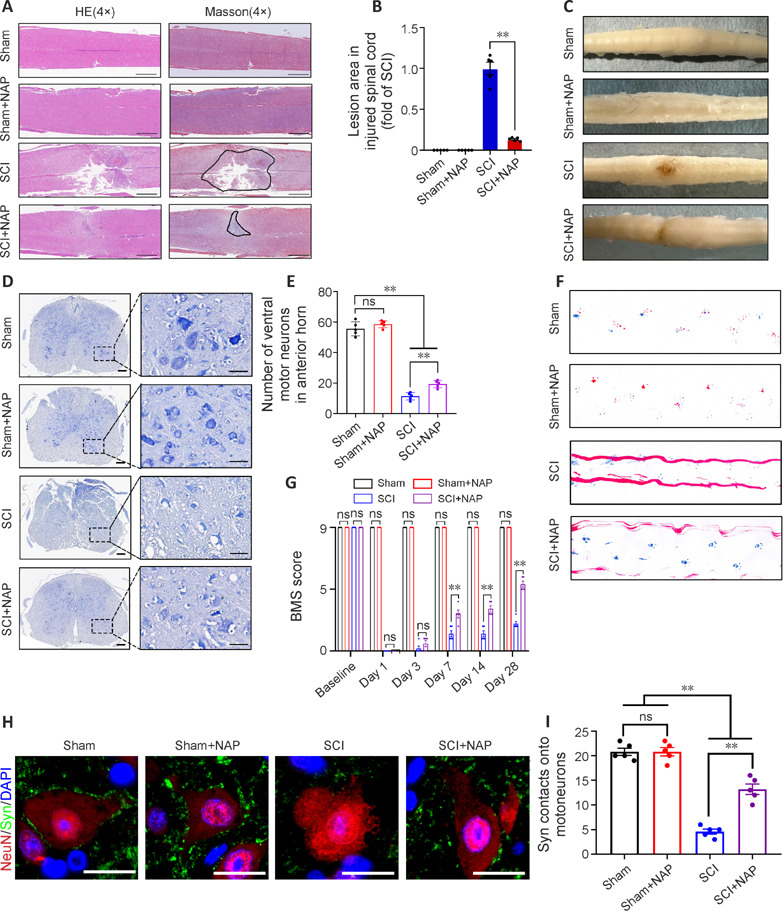
NAP promotes locomotor function recovery in mice after SCI. (A) Longitudinal spinal cord sections from the Sham, Sham + NAP, SCI, and SCI + NAP groups at 28 days were examined via HE and Masson staining (scale bars: 400 μm). (B) Masson-positive lesion sizes within the spinal cords of mice from the respective groups. (C) Spinal cord sections from mice in the Sham, Sham + NAP, SCI, and SCI + NAP groups 28 days after surgery. (D) Cross-sections of spinal cords from mice from each group at 28 days were subjected to Nissl staining. Scale bars: 100 μm on the left and 25 μm on the right. (E) Number of ventricle motor neurons in spinal cord cross-sections from the indicated groups. (F) Gait analysis for each group at 28 days. Blue: Forepaw print; red: hindpaw print. (G) Basso mouse scale (BMS) scores for the indicated groups at baseline and 1, 3, 7, 14, and 28 days. (H) Spinal cord sections stained with Syn (green) and NeuN (red). Scale bars: 25 μm. (I) Number of positive synapses in each group. Data are shown as the mean ± SEM. *n* = 5. **P < 0.01. BMS: Basso Mouse Scale; DAPI: 4′,6-diamidino-2-phenylindole; HE: hematoxylin-eosin; NAP: davunetide; ns: not significant; SCI: spinal cord injury; SYN: synaptophysin.

Initially, we focused on assessing NAP’s neuroprotective effects post-SCI. Masson, HE, and Nissl staining revealed a marked reduction in spinal cord lesion areas and neuronal loss in the SCI + NAP group compared with the SCI group (*P* < 0.01; **Figure 2A**, **B**, **D**, and **E**). Additionally, the spinal cord samples depicted in **Figure 2C** demonstrated smaller lesion areas compared with the NAP-treated group. IF staining for GFAP in spinal cord sections showed the formation of a glial scar post-injury, and NAP treatment decreased glial scar formation compared with the SCI group (*P* < 0.01; **Additional Figure 1A** and **B**). Co-immunofluorescence staining highlighted the interaction of neural synapses (Syn) with neurons in the spinal cord. At 28 days post-injury, IF staining showed a decreased in the number of Syn-positive synapses in the SCI group compared with the Sham and Sham + NAP groups, and this effect was reversed by NAP administration (*P* < 0.01 for all; **Figure 2H** and **I**), suggesting that NAP treatment promoted the recovery of neuronal synapses. IF staining for NF200 to detect axonal regeneration showed that the density of regenerated axons in the SCI + NAP group was significantly higher than that in the SCI group, indicating that NAP treatment significantly promoted axon regeneration after SCI (*P* < 0.01; **Additional Figure 1C** and **D**). Moreover, there was a significant decrease in BMS scores and disruption in the continuity of hind limb footprints in SCI mice after 28 days, pointing to severe impairment in hind limb movement. However, NAP treatment mitigated these effects (**Figure 2F** and **G**), indicating that NAP promotes motor function recovery following SCI. These results indicate that NAP has a neuroprotective effect and promotes motor function recovery.

### Davunetide inhibits neuronal necroptosis after spinal cord injury

**Figure 3:**
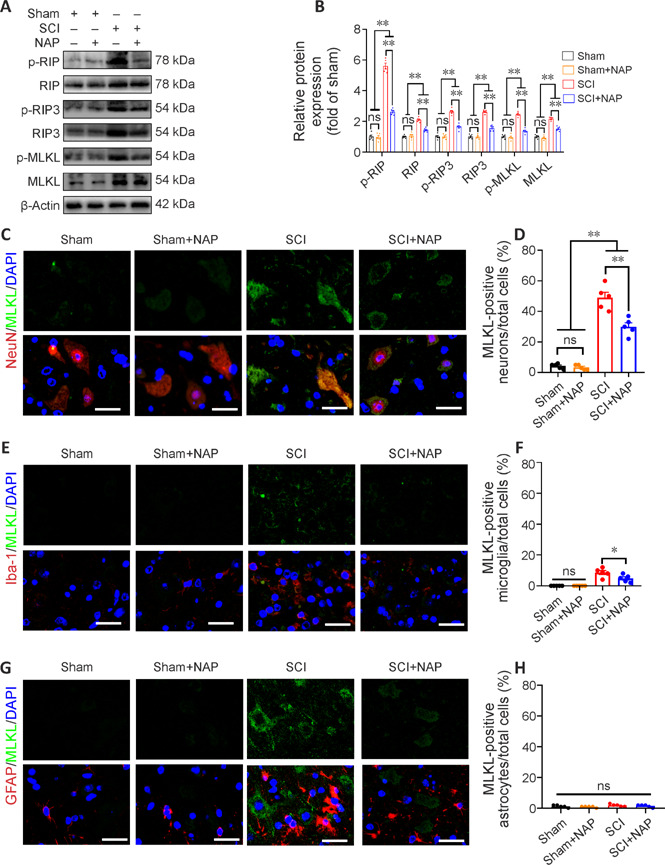
NAP inhibits neuronal necroptosis after SCI. (A) Western blot analysis of RIP, p-RIP, RIP3, p-RIP3, MLKL, and p-MLKL expression levels in the Sham, Sham + NAP, SCI, and SCI + NAP groups. β-Actin was utilized as a loading control. (B) Quantification of RIP, p-RIP3, RIP3, p-RIP3, MLKL, and p-MLKL expression levels in each group. (C) Double immunofluorescence staining for MLKL (green) and NeuN (red) in the spinal cords of mice from the Sham, Sham + NAP, SCI, and SCI + NAP groups. (D) Ratio of MLKL-positive neurons to total cells. (E) Double immunofluorescence staining for MLKL (green) and Iba-1 (red) in the spinal cords of mice from the Sham, Sham + NAP, SCI, and SCI+NAP groups. (F) Ratio of MLKL-positive microglial cells to total cells. (G) Double immunofluorescence staining for MLKL (green) and GFAP (red) in the spinal cords of mice from the Sham, Sham + NAP, SCI, and SCI+NAP groups. Scale bars: 25 μm in C, E, and G. (H) Ratio of MLKL-positive astrocytes to total cells. Data are shown as the mean ± SEM. *n* = 5. *P < 0.05, **P < 0.01. DAPI: 4′,6-Diamidino-2-phenylindole; GFAP: glial fibrillary acidic protein; Iba-1: ionized calcium-binding adapter molecule 1; MLKL: mixed lineage kinase domain-like protein; NAP: davunetide; ns: not significant; RIP: receptor-interacting protein kinase; SCI: spinal cord injury.

Following SCI, necroptosis exacerbates spinal cord tissue damage. Mitigating necroptosis is therefore vital for spinal cord recovery post-SCI. We explored NAP’s potential to inhibit necroptosis by analyzing the expression of necroptosis markers such as p-RIP, RIP, phosphorylated RIP3 (p-RIP3), RIP3, phosphorylated MLKL (p-MLKL), and MLKL. NAP treatment did not have a significant effect on necroptosis marker expression levels in the absence of SCI (Sham *vs*. Sham + NAP, **Figure 3A** and **B**). However, there was a significant elevation in necroptosis marker expression in the SCI group compared with the Sham and Sham + NAP groups (*P* < 0.01), and treatment with NAP reversed this effect (**Figure 3A** and **B**). Post-SCI, astrocytes contribute to glial scar formation, while microglia facilitate neuroinflammation (Anjum et al., 2020). Co-immunofluorescence (co-IF) staining performed on day 3 post-SCI (**Figure 3C–H**) showed that MLKL predominantly accumulated in neurons, rather than microglia or astrocytes, signifying that necroptosis primarily occurred in this cell type (**Figure 3C** and **D**). Notably, the fluorescence intensity of MLKL in the neurons in the SCI group was substantially higher than that in the Sham and Sham + NAP groups at 3 days post-SCI (*P* < 0.01; **Figure 3C** and **D**). NAP treatment reduced neuronal MLKL fluorescence intensity (**Figure 3C** and **D**), suggesting that NAP inhibits neuronal necroptosis following SCI. These results indicated that NAP mitigates neuronal necroptosis after SCI.

### Davunetide enhances neural autophagy after spinal cord injury

**Figure 4:**
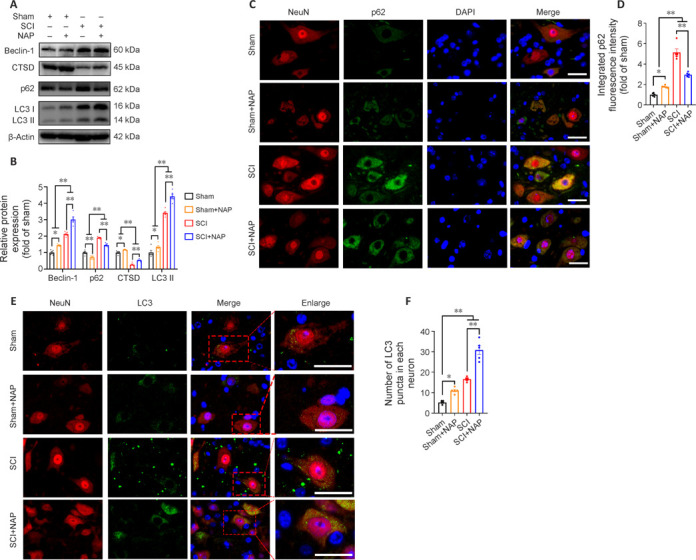
NAP promotes neural autophagy after SCI. (A) Western blot bands of Beclin-1, CTSD, p62, and LC3 in the Sham, Sham + NAP, SCI, and SCI + NAP groups. β-Actin was utilized as a loading control. (B) Quantification of Beclin-1, CTSD, p62, and LC3 II protein expression levels in each group. (C) Double immunofluorescence staining for p62 (green) and NeuN (red) in the spinal cords of mice from the Sham, Sham + NAP, SCI, and SCI + NAP groups. (D) p62 immunofluorescence density in each group. (E) Double immunofluorescence staining for LC3 (green) and NeuN (red) in the spinal cords of mice from the Sham, Sham + NAP, SCI, and SCI + NAP groups. Scale bars: 25 μm in C and E. (F) Number of LC3 puncta in each neuron. Data are shown as the mean ± SEM. *n* = 5. *P < 0.05, **P < 0.01. CTSD: Cathepsin D; DAPI: 4′,6-diamidino-2-phenylindole; LC3: light chain 3; NAP: davunetide; NeuN: neuron-specific nuclear protein; ns: not significant.

Prior research has established autophagy as a critical mechanism for SCI repair, as it eliminates damaged organelles or proteins (Abbaszadeh et al., 2020; Jiang et al., 2022). Notably, the autophagy-lysosome pathway can degrade necroptosis-related effector proteins such as RIPK1, while inhibiting autophagy post-SCI impedes the degradation of necroptosis markers (Liu et al., 2018). To assess the influence of NAP on autophagy in the context of SCI, we conducted WB and IF analyses. Compared with the Sham group, the levels of Beclin-1, LC3-II, and CTSD in the Sham + NAP group slightly increased, while the levels of p62 decreased, in both the WB and IF analyses (*P* = 0.014, 0.0382, 0.0197 and 0.0032, respectively; **Figure 4A–F**). However, 3 days post-injury, the autophagy markers Beclin-1 and LC3 II were significantly elevated in the SCI group compared with the Sham group, alongside a notable increase in the autophagy substrate p62 (**Figure 4A** and **B**). Additionally, expression of the autolysosomal biomarker CTSD, which was reduced post-SCI, increased following NAP treatment (**Figure 4A** and **B**). Post-NAP treatment, an increase in autophagy markers and a decrease in p62 were observed in SCI mice, indicating that NAP promotes autophagy post-SCI. Co-IF analysis showed LC3 and p62 expression in neurons in the Sham, Sham + NAP, SCI, and SCI + NAP groups (**Figure 4C–F**). Treatment with NAP increased LC3 expression and decreased p62 expression compared with the SCI group (*P* < 0.01). Aligning with the WB findings, the IF results suggested that NAP treatment increased neuronal autophagy levels post-SCI. These findings collectively demonstrate NAP’s role in enhancing autophagy following SCI.

### Inhibiting autophagy reverses the negative effects of davunetide on neuronal necroptosis after spinal cord injury

**Figure 5:**
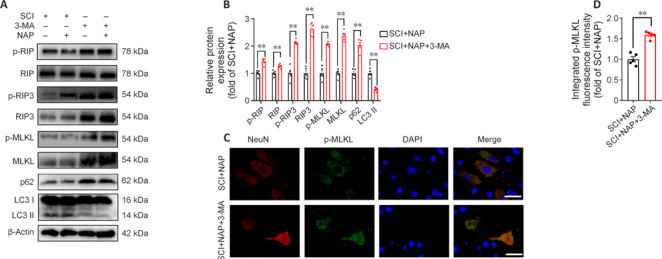
Blocking autophagy with 3MA counteracts the ability of NAP to inhibit neuronal necroptosis post-SCI. (A) Western blot analyses of RIP, p-RIP, RIP3, p-RIP3, MLKL, p-MLKL, p62, and LC3 protein expression in spinal cord lesions. β-Actin was utilized as a loading control. (B) Quantification of RIP, p-RIP, RIP3, p-RIP3, MLKL, p-MLKL, p62, and LC3II expression in each group. (C) Double immunofluorescence staining for p-MLKL (green) and NeuN (red) in the spinal cords of mice from the SCI + NAP and SCI + NAP + 3MA groups. Scale bar: 25 μm. (D) p-MLKL immunofluorescence density. Data are shown as the mean ± SEM. *n* = 5. **P < 0.01. 3MA: 3-Methyladenine; DAPI: 4′,6-diamidino-2-phenylindole; LC3: light chain 3; MLKL: mixed lineage kinase domain-like protein; NAP: davunetide; NeuN: neuron-specific nuclear protein; ns: not significant; RIP: receptor-interacting protein kinase; SCI: spinal cord injury.

To elucidate the interplay between autophagy and necroptosis, we used 3-MA, a selective PI3K inhibitor, to hinder autophagosome formation (Wu et al., 2010). In comparison with the SCI + NAP group, the addition of 3MA decreased LC3 II expression and increased p62 expression (*P* < 0.01), indicating inhibition of autophagy. In addition, treatment with 3MA led to a notable increase in the protein levels of the necroptosis markers p-RIPK1, RIPK1, p-RIPK3, RIPK3, p-MLKL, and MLKL, in the SCI + NAP + 3-MA group in comparison with the SCI + NAP group (*P* < 0.01 for all; **Figure 5A** and **B**). Additionally, IF analysis of MLKL expression revealed a significant enhancement in MLKL fluorescence intensity in neurons in the SCI + NAP + 3-MA group compared with the SCI + NAP group (*P* < 0.01; **Figure 5C** and **D**). These results suggest that NAP alleviates neuronal necroptosis by enhancing autophagy, indicating the important role of autophagy in NAP-mediated neuroprotection. These findings suggest that NAP mitigates neural necroptosis by augmenting autophagy, and that inhibiting autophagy diminishes NAP’s capacity to counteract necroptosis.

### Davunetide promotes SIRT1-induced autophagy to inhibit neuronal necroptosis after spinal cord injury

**Figure 6:**
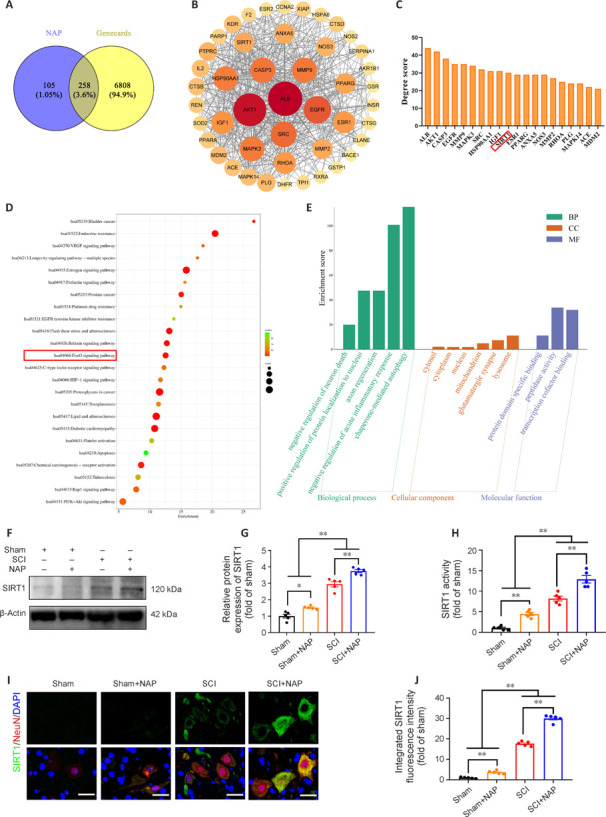
NAP promotes SIRT1-induced autophagy, thereby inhibiting neuronal necroptosis after SCI. (A) Venn diagram showing overlapping SCI target genes and predicted NAP target genes. (B) A protein–protein interaction diagram exhibiting the distribution of potential NAP targets. (C) Histogram of the top 20 potential NAP targets. (D, E) The top 20 enriched KEGG pathways and GO analysis of the overlapping gene set shown in A. (F) Western blot bands of SIRT1 in the spinal cord of mice from the Sham, Sham + NAP, SCI and SCI + NAP groups. β-Actin was utilized as a loading control. (G) SIRT1 protein expression levels shown in F. (H) SIRT1 activity in the spinal cord of mice from each group. (I) Double immunofluorescence staining for SIRT1 (green) and NeuN (red) in the spinal cords of mice from the Sham, Sham + NAP, SCI, and SCI + NAP groups. Scale bars: 25 μm. (J) Quantification of SIRT1immunofluorescence density. Data are shown as the mean ± SEM. *n* = 5. *P < 0.05, **P < 0.01. DAPI: 4′,6-Diamidino-2-phenylindole; NAP: davunetide; NeuN: neuron-specific nuclear protein; ns: not significant; SCI: spinal cord injury; SIRT1: Sirtuin1.

**Figure 7:**
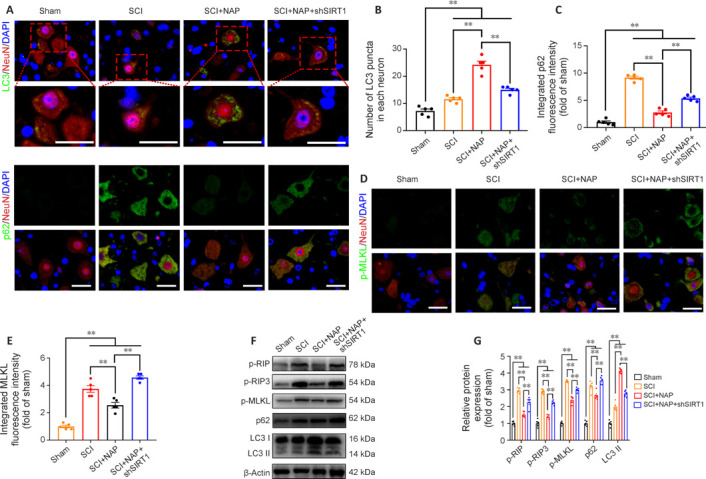
SIRT1 knockdown reversed NAP-mediated promotion of neural autophagy and inhibition of neural necroptosis after SCI. (A) Immunofluorescence staining image for p62 (green) with NeuN (red) and LC3 (green) with NeuN (red) in the spinal cords of mice from the Sham, SCI, SCI + NAP, and SCI + NAP + shSIRT1 groups on day 3 after SCI. (B) Number of LC3 puncta in each neuron from each group. (C) Quantification of p62 immunofluorescence density in each group. (D) Immunofluorescence staining for p-MLKL (green) with NeuN (red) in the spinal cords of mice from the Sham, SCI, SCI + NAP, and SCI + NAP+ shSIRT1 groups on day 3 after SCI. Scale bars: 25 μm in A and D. (E) Quantification of p-MLKL immunofluorescent density in each group. (F) Western blot bands of p-RIP, p-RIP3, p-MLKL, p62, and LC3 in the spinal cord lesion on day 3 after SCI. β-Actin was utilized as a loading control. (G) p-RIP, p-RIP3, p-MLKL, p62, and LC3 II protein expression levels were quantified in each group. Statistical analysis was performed using two-way analysis of variance followed by Tukey’s multiple comparisons test. Data are shown as the mean ± SEM. *n* = 5. **P < 0.01. DAPI: 4′,6-Diamidino-2-phenylindole; LC3: light chain 3; MLKL: mixed lineage kinase domain-like protein; NAP: davunetide; NeuN: neuron-specific nuclear protein; RIP: receptor-interacting protein kinase; SCI: spinal cord injury.

Next, network pharmacology analysis was conducted to determine the mechanism by which NAP regulates neuronal autophagy. Targets downstream of NAP were gathered from the Pharmapper and Swiss Target Prediction databases, and SCI-related disease targets were collected from GeneCards. Comparing these listed of targets yielded 258 common targets (**Figure 6A**). A protein–protein interaction network was constructed for these targets using Cytoscape software, which identified 15 targets with higher interaction scores (**Figure 6B** and **C**). Among these, SIRT1, a deacetylase, was of particular interest. Previous studies have indicated that SIRT1 facilitates autophagy progression through various signaling pathways (Deng et al., 2021; Chen et al., 2022), suggesting that NAP might regulate autophagy via SIRT1 modulation. Thus, we examined SIRT1 expression and found that it is increased post-SCI, and that this effect was enhanced by NAP treatment (**Figure 6F** and **G**). Additionally, NAP treatment enhanced SIRT1 activity in comparison with the SCI group (*P* < 0.01; **Figure 6H**). IF staining demonstrated SIRT1 expression in neurons post-SCI (**Figure 6I** and **J**), with the SCI+NAP group showing significantly higher SIRT1 fluorescence density compared with the other groups (*P* < 0.01). Next, shRNA-mediated SIRT1 knockdown in the mouse spinal cord was performed, followed by a series of rescue experiments. LC3II levels were decreased and autophagy substrate p62 and necroptosis marker (p-RIPK1, RIPK1, p-RIPK3, RIPK3, p-MLKL, and MLKL) levels were increased in the SCI+NAP+shSIRT1 group compared with the SCI + NAP group (*P* < 0.01 for all; **Figure 7F** and **G**). The reason for the decrease in LC3 II levels after SIRT1 knockdown may be related to the reduced regulation of autophagy genes by SIRT1. IF analysis showed greater numbers of LC3 fluorescent puncta in neurons from the SCI + NAP group compared with the SCI and Sham groups, while p62 and MLKL fluorescence density was lower in the SCI + NAP group compared with the SCI group (*P* < 0.01 for all; **Figure 7A–E**). However, these effects were reversed following SIRT1 knockdown. Collectively, these findings suggest that NAP enhances neuronal autophagy and inhibits necroptosis by modulating SIRT1.

### Davunetide promotes motor function recovery after spinal cord injury by regulating SIRT1

We investigated the impact of SIRT1 knockdown on the ability of NAP to promote motor function recovery. Masson and HE staining showed that NAP treatment led to a reduction in the spinal cord injury area in mice, and this effect was counteracted by SIRT1 knockdown (**Additional Figure 2A** and **B**). Nissl staining further indicated a substantial decrease in the number of anterior horn neurons in the SCI + NAP + shSIRT1 group compared with the SCI + NAP group (*P* < 0.01; **Additional Figure 2D** and **E**). IF staining revealed a reduced number of neural synapses in mice subjected to SIRT1 knockdown and treated with NAP compared with the NAP-only group (**Additional Figure 2H** and **I**). Behavioral experiments demonstrated that SIRT1 knockdown significantly negated the beneficial effects of NAP on motor function recovery post-SCI (**Additional Figure 2F** and **G**). Collectively, these findings suggest that NAP promotes motor function recovery post-SCI by regulating SIRT1.

### Davunetide promotes neural autophagy after spinal cord injury through the SIRT1-FOXO1-TFEB axis

**Figure 8:**
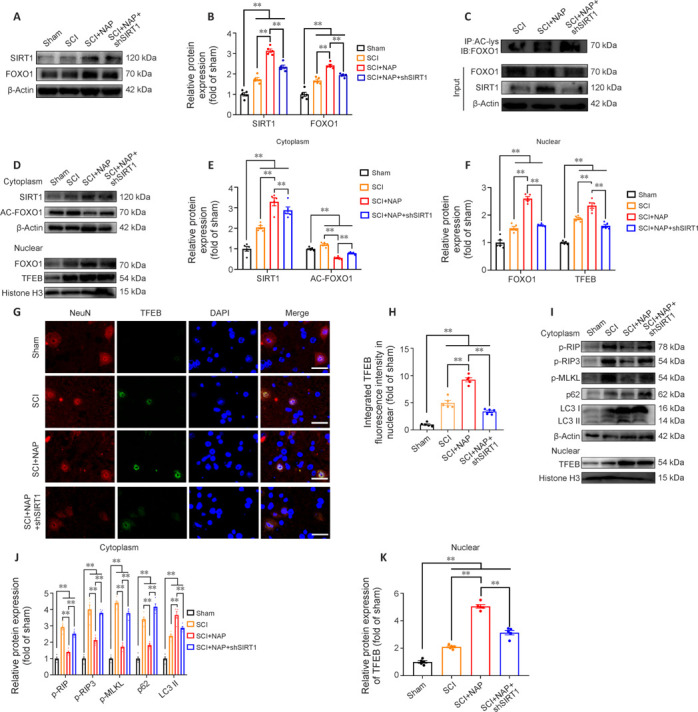
NAP promotes neural autophagy after SCI through the SIRT1-FOXO1-TFEB pathway. (A) Western blot bands of SIRT1 and FOXO1 in the spinal cords of mice from the Sham, SCI, SCI + NAP, and SCI + NAP + shSIRT1 groups. β-Actin was utilized as a loading control. (B) Quantification of SIRT1 and FOXO1 expression levels in each group. (C) FOXO1 acetylation levels in the spinal cords of mice from the SCI, SCI + NAP, and SCI + NAP + shSIRT1 groups, as assessed by immunoprecipitation with an anti-acetyl-lysine antibody followed by immunoblotting with a FOXO1 antibody. (D) Western blot analysis of SIRT1, cytoplasmic AC-FOXO1 and FOXO1, and nuclear TFEB expression in the spinal cords of mice from the Sham, SCI, SCI + NAP, and SCI + NAP + shSIRT1 groups. β-actin was utilized as a loading control for the cytoplasmic proteins, and histone H3 was utilized as a loading control for the nuclear proteins. (E) Quantification of SIRT1 and AC-FOXO1 expression levels in the cytoplasm in each group. (F) Quantification of TFEB and FOXO1 expression levels in the nuclei in each group. (G) Immunofluorescence staining for TFEB (green) with NeuN (red) in the spinal cords of mice from the Sham, SCI, SCI + NAP, and SCI + NAP + shTFEB groups on day 3 after SCI. Scale bars: 25 μm. (H) Quantification of nuclear TFEB immunofluorescence density in each group. (I) Western blot analysis of cytoplasmic p-RIP, p-RIP3, p-MLKL, p62, and LC3 expression and nuclear TFEB expression in the spinal cords of mice from the Sham, SCI, SCI + NAP, and SCI + NAP + shSIRT1 groups. β-Actin was utilized as a loading control for cytoplasmic proteins, and histone H3 was utilized as a loading control for nuclear proteins. (J) p-RIP, p-RIP3, p-MLKL, p62 and LC3 II protein expression levels in the cytoplasm were quantified in each group. (K) TFEB expression levels in the nucleus were quantified in each group. **P < 0.01. DAPI: 4′,6-Diamidino-2-phenylindole; FOXO1: forkhead box protein O1; LC3: light chain 3; MLKL: mixed lineage kinase domain-like protein; NAP: davunetide; NeuN: neuron-specific nuclear protein; RIP: receptor-interacting protein kinase; SCI: spinal cord injury; TFEB: transcription factor EB.

KEGG analysis identified signaling pathways potentially modulated by NAP; the FOXO pathway was of particular interest (**Figure 6D**). **Figure 6E** shows the main secondary GO annotations O, which showed the effects of NAP on biological processes, cell components, and molecular functional analysis after SCI. SIRT1 deacetylates FOXO1 to regulate autophagy (Hariharan et al., 2010; Ren et al., 2020; Luchetti et al., 2022), suggesting that NAP may influence autophagy via the SIRT1-FOXO1 axis. Western blot analysis demonstrated an upregulation in FOXO1 expression following SCI that was further augmented by NAP treatment (**Figure 8A** and **B**). However, SIRT1 knockdown impeded the effects of NAP on FOXO1. Immunoprecipitation analysis showed that NAP treatment decreased FOXO1 acetylation, and that this effect was reversed by SIRT1 knockdown (**Figure 8C**). FOXO1 has been reported to enhance the expression of TFEB, which is associated with autophagy regulation, by binding to its promoter (Tao et al., 2021). We observed reduced cytoplasmic acetylated FOXO1 and increased nuclear FOXO1 and TFEB post-NAP treatment compared with the SCI group **Figure 8D–F**), and found that these effects were decreased by SIRT1 knockdown. Immunofluorescence analysis demonstrated higher TFEB intensity in SCI + NAP group neurons than in neurons in the SCI group, and this effect was reduced by SIRT1 knockdown (**Figure 8G** and **H**). Furthermore, a decrease in autophagy and an increase in necroptosis were observed in the SCI + NAP + shTFEB group, suggesting a reversal of the effects of NAP (**Figure 8I–K**). These findings suggest that NAP enhances neuronal autophagy and inhibits necroptosis post-SCI by promoting SIRT1-FOXO1-TFEB signaling.

## Discussion

Recent research on SCI treatment has focused on cell transplantation, spinal cord electrical stimulation, mitigation of secondary neuronal death, and promotion of axonal regeneration (Karamian et al., 2022; Wang and Bai, 2024; Mittal et al., 2025; Olaya et al., 2025). Recent advances also highlight the crucial role of the brain–spine interface in motor function recovery in SCI patients, positioning it as a potential future focus of SCI treatment strategies (Lorach et al., 2023). However, cell transplantation poses significant risks, including potential cancer development, and remains uncertain, while the brain–spine interface, despite its potential, requires extensive research and involves substantial costs (Jeong et al., 2011). Consequently, pharmacological interventions continue to be a primary approach for SCI repair. Proteins such as growth factors have been identified that facilitate neuronal and motor function recovery post-SCI (Xu et al., 2021; Li et al., 2024). The results from our study indicate that a peptide fragment called NAP, derived from ADNP, has significant potential to promote motor function recovery and provide neuroprotection after SCI. We found that NAP activated SIRT1, thereby deacetylating FOXO1, leading to FOXO1 transfer from the cytoplasm to the nucleus, subsequently upregulating TFEB expression, after SCI. Furthermore, of the upregulation in TFEB expression promoted autophagy and inhibited necroptosis, providing a positive driving force for recovery from SCI. Thes findings have significant clinical implications, as autophagy clears abnormal proteins and organelles from cells, promoting cellular repair and regeneration. Based on our findings, NAP is promising therapeutic agent, offering new directions and possibilities for the treatment of SCI.

Previous studies have highlighted the involvement of necroptosis in post-SCI pathophysiology (Liu et al., 2018; Hu et al., 2022). We observed a marked increase in the expression of necroptosis markers post-SCI, indicating increased necroptosis in neurons after SCI. Remarkably, NAP suppressed neural necroptosis after injury. This aligns with earlier research suggesting that inhibiting necroptosis can protect neuronal function and foster spinal cord recovery (Xu et al., 2023a, b). Therefore, our findings suggest that NAP plays a significant role in mitigating necroptosis in SCI, contributing to recovery.

To identify the mechanisms by which NAP mitigates necroptosis post-SCI, we examined autophagy, a process known to be neuroprotective following SCI. Studies have suggested that autophagy, which is responsible for the removal of damaged proteins and organelles, thus maintaining cellular metabolic balance, is crucial for functional recovery after SCI (Wu et al., 2021; Li et al., 2022; Xu et al., 2023b). We found that NAP significantly enhanced neural autophagy. Treatment with 3MA, an autophagy inhibitor, reversed NAP’s effects, including promotion of on function recovery and inhibition of necroptosis. Consistent with previous studies, this result suggests that NAP exerts its therapeutic effects post-SCI by promoting autophagy, which in turn contributes to the suppression of necroptosis in neurons.

NAP, an eight-amino-acid peptide, has demonstrated significant neuroprotective properties in both *in vitro* and *in vivo* studies (Gozes, 2011). Clinical trials have shown that NAP enhances memory in patients with amnestic mild cognitive impairment and improves daily behavior in patients with schizophrenia (Gozes, 2011). Considering the neuroprotective role of NAP in neurodegenerative diseases, we hypothesized that it would have a protective effect on SCI. A study of schizophrenia pathophysiology revealed that the reduction in beclin-1 expression, which correlated with ADNP dysregulation, was countered by treatment with NAP, which stabilized LC3 and augmented autophagy (Merenlender-Wagner et al., 2014). Consistent with this, we found that NAP promotes autophagy. In addition, our network pharmacology analysis suggested that NAP’s positive impact on SCI might be linked to SIRT1 activation. Western blot and immunofluorescence analyses confirmed that NAP enhanced autophagy via the SIRT1-FOXO1-TFEB regulatory pathway. TFEB, which is crucial for lysosome biogenesis, facilitates autophagosome formation and fusion with lysosomes (Mansueto et al., 2017; Mao et al., 2020). A previous study showed that TFEB upregulation leads to increased expression of autophagy-related genes and initiation of autophagy (Xu et al., 2023b). Our result indicate that NAP promotes autophagy via TFEB, as previously reported, and that SIRT1-FOXO1-TFEB signaling activates autophagy, thereby inhibiting necroptosis and promoting neural repair. Thus, NAP regulates autophagy in a TFEB-dependent manner, providing new insights into potential treatment approaches for central nervous system trauma such as SCI and traumatic brain injury.

This study has some limitations that should be noted. First, the network pharmacology analysis suggested the involvement of pathways such as PI3K and MAPK in the action of NAP, and a prior study indicated that NAP regulates these pathways to reduce neuronal apoptosis (Gozes, 2011). Yet, their specific roles in SCI require additional investigation. Second, our study primarily focused on the early stages of autophagy and did not assess autophagy flux. Inhibition of autophagic flux, which is critical in SCI, merits further exploration (Zhou et al., 2020). This flux is often linked to lysosomal dysfunction (Liu et al., 2018). Future experiments should address this aspect. Third, we delivered NAP administration primarily via nasal injection, which raises questions about its effective delivery to the lesion site. Exploring alternative delivery methods, such as mixing NAP with hydrogels for direct application to the injured spinal cord area, may prove beneficial.

In summary, our findings demonstrate that NAP substantially enhances motor function recovery following SCI. This improvement is attributed to promotion of autophagy, which in turn inhibits necroptosis. The increase in autophagy is facilitated by SIRT1-mediated deacetylation of FOXO1, leading to its nuclear translocation and subsequent stimulation of TFEB expression. These results collectively provide encouraging evidence for NAP’s therapeutic potential in SCI treatment. Looking ahead, further studies focused on advancing the clinical application of NAP for patients with SCI are highly anticipated.

## Additional files:

***Additional Figure 1:*** *NAP promotes axon regeneration and decreases glial scar formation after SCI.*

Additional Figure 1 NAP promotes axon regeneration and decreases glial scar formation after SCI.
(A) Longitudinal spinal cord sections from each group stained for GFAP (green). White dotted outlines indicate areas of glial scar. Scale bars: 50 μm. (B) Quantification of glial scar area within the spinal cord of mice from the indicated groups. (C) Spinal cord sections from mice in the indicated groups stained for NF200 (green) and NeuN (red). Scale bars: 50 μm. (D) Number of NF200-positive fluorescent puncta in each group. *P < 0.05, **P < 0.01. DAPI: 4 ′, 6-Diamidino-2-phenylindole; GFAP: glial fibrillary acidic protein; NAP: davunetide; NeuN: neuron-specific nuclear protein; NF200: Neurofilament heavy polypeptide; SCI: spinal cord injury.

***Additional Figure 2:*** *SIRT1 knockdown counteracts the neuroprotective effects of NAP after SCI.*

Additional Figure 2 SIRT1 knockdown counteracts the neuroprotective effects of NAP after SCI.
(A) Longitudinal spinal cord sections from mice in the Sham, SCI, SCI + NAP, and SCI + NAP + shSIRT1 groups at 28 days were examined via HE and Masson staining. Scale bars: 400 μm. Black circles indicate the injured areas. (B) Quantification of Masson-positive lesions in the spinal cords of mice from each group. (C) Spinal cord sections from mice in the Sham, SCI, SCI+NAP, and SCI+NAP+shSIRT1 groups 28 days after surgery. (D) Spinal cord cross-sections from mice in the indicated groups at 28 days after surgery were examined via Nissl staining. Scale bars: 100 μm on the left and 25 μm on the right. (E) Number of ventricle motor neurons in the spinal cord cross-sections from each group. (F) Gait analysis for each group 28 days after surgery. Blue: forepaw print; red: hindpaw print. (G) Basso Mouse Scale (BMS) scores for the indicated groups at baseline and on days 1, 3, 7, 14, and 28. (H) Spinal cord sections from mice in the indicated groups stained with Syn (green) and NeuN (red). Scale bars: 25 μm. (I) Number of positive synapses in each group. Data are shown as the mean ± SEM. n = 5. **P < 0.01. BMS: Basso Mouse Scale; DAPI: 4′,6-diamidino-2-phenylindole; HE: hematoxylin-eosin; NAP: davunetide; NeuN: neuron; ns: not significant; SCI: spinal cord injury; SYN: synapse.

## Data Availability

*All relevant data are within the paper and its Additional files.*
